# Assessment of shoulder function using the coronal plane angle

**DOI:** 10.4103/0973-6042.63217

**Published:** 2009

**Authors:** N. D. Clement, M. Fuller, R. C. Colling, A. N. Stirrat

**Affiliations:** Department of Orthopedics and Trauma, Sunderland Royal Hospital, SR4 7TP

**Keywords:** Shoulder, Constant score, Oxford score, objective, assessment, coronal plain

## Abstract

**Background:**

Assessment of shoulder function is an essential part of clinical practice. Current scoring relies on multiple subjective and / or objective components. We present a single angular measurement, the coronal plane angle, which relates to the functional assessment of the shoulder.

**Materials and Methods:**

One hundred patients were prospectively enrolled and assessed using the Constant-Murley score and the Oxford shoulder questionnaire, and the coronal plane angle was measured for both symptomatic and asymptomatic shoulder.

**Results:**

Nine patients were excluded from the study: Four had apprehension and five were not able to get their hand to head. The mean coronal plane angle on the symptomatic side was +11.3° and the asymptomatic side −1.5° (*P* ≤ 0.01). Pearson's correlation of 0.9 and 0.84 was demonstrated for the Constant-Murley and Oxford shoulder scores, respectively, with the coronal plane angle.

**Conclusion:**

The coronal plane angle is a single objective assessment and provides a simple alternative to shoulder assessment for the majority of patients.

## INTRODUCTION

Clinical practice and research depend on the outcome measures, to evaluate the efficacy of treatment.[1] Constant-Murley shoulder score[2] and Oxford shoulder score[3] are assessment tools and are applicable to most patients. Scoring includes time-consuming angular measurements in several planes as part of the objective assessment. To our knowledge, no other published article has described the use of a single angular measurement for the objective assessment of the shoulder and the association with commonly used scoring tools. The aim of this study is to ascertain the correlation between a single composite angular measurement with both subjective and objective assessments of shoulder function.

## MATERIALS AND METHODS

**Figure 1:**
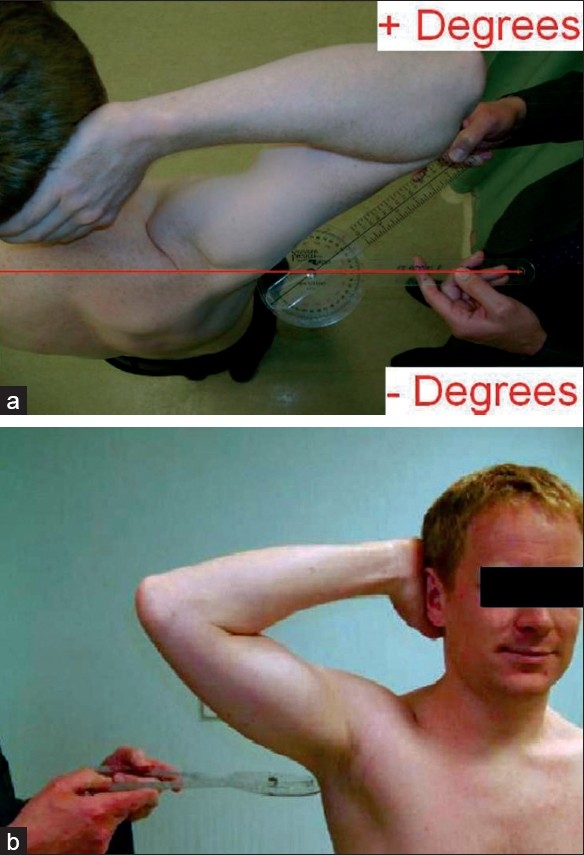
(a, b) Measuring the coronal plain angle

A prospective study was conducted over a period of 12 months. Neutral observers (NC, MF) assessed patients presenting with shoulder symptoms to the new patient clinic of the senior author. The Constant-Murley score was recorded excluding the power component, thus the maximum possible score was 75. The patients were also asked to complete an Oxford Shoulder questionnaire. In addition the patients were asked to place a hand behind their head and push the elbow as far posterior as possible [Figure 1a], whereupon, the “coronal plane” angle was measured between the upper arm and the coronal plane with a goniometer held in the transverse plane [Figure 1b]. This angle was measured twice by the same observer for both symptomatic and asymptomatic shoulders, before and after the objective measures for the Constant score, and a mean value was taken.

Patients with apprehension due to recurrent instability and those who were unable to actively place hand to head were excluded. For those patients who could not physically get their hand to head Constant scores and Oxford Shoulder scores (OSS) were completed, but they were excluded from the correlation analysis. A clinical diagnosis of the shoulder pathology was recorded for each, which was assigned by the senior author.

A Pearson's correlation analysis was performed between the totals and components of the Constant score and OSS with the coronal plane angle. A Mann-Whitney U test was used to compare the symptomatic and asymptomatic coronal plane angles.

## RESULTS

One hundred patients were enrolled, of which there were 104 symptomatic shoulders. The mean age was 53.3 years. Fifty-four were female. Nine patients were excluded from the study: four had apprehension and five were not able to get hand to head. Of the latter group all had a Constant score of less than 10 and an OSS of greater than 55.

**Figure 2:**
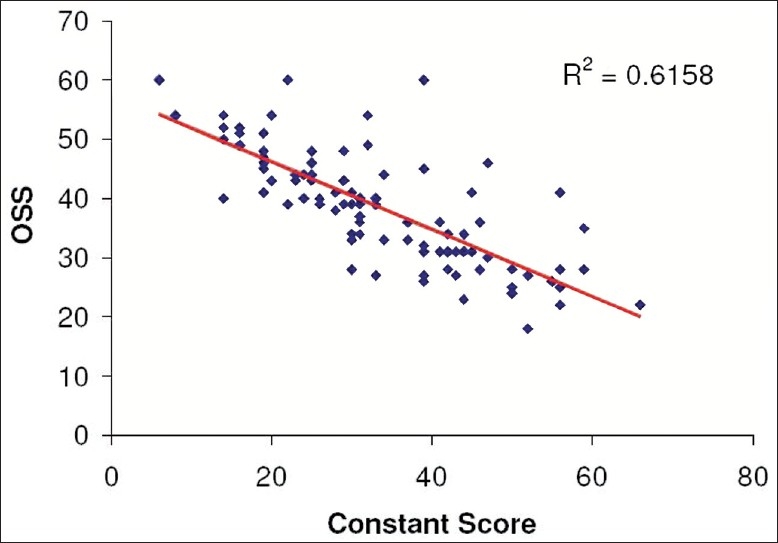
Correlation of OSS with Constant-Murley score

The mean coronal plane angle on the symptomatic side was +11.30 (range: 00 to 750) and the asymptomatic side −1.50 (range: -300 to 300) (*P* ≤ 0.01). Mean Constant and OSS were 33.8 and 38.4/60, respectively. Pearson's correlation of 0.90 was demonstrated between these scores [Figure 2].

**Figure 3:**
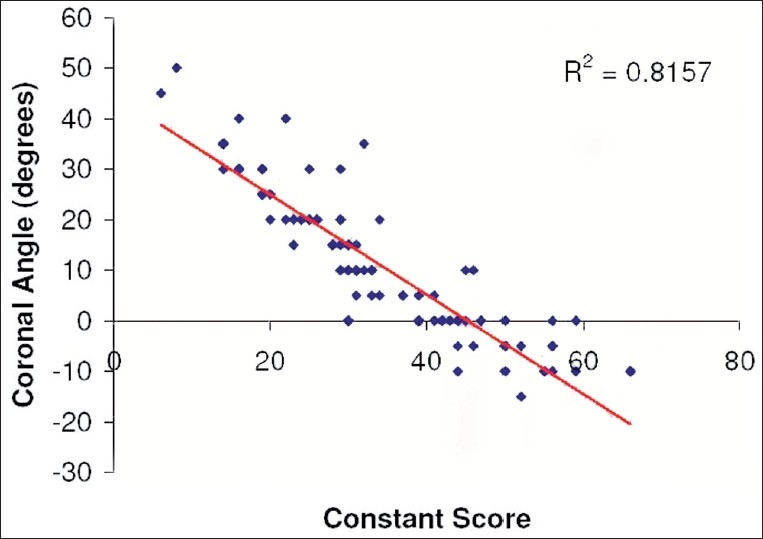
Correlation of the coronal plane angle with the Constant score

**Figure 4:**
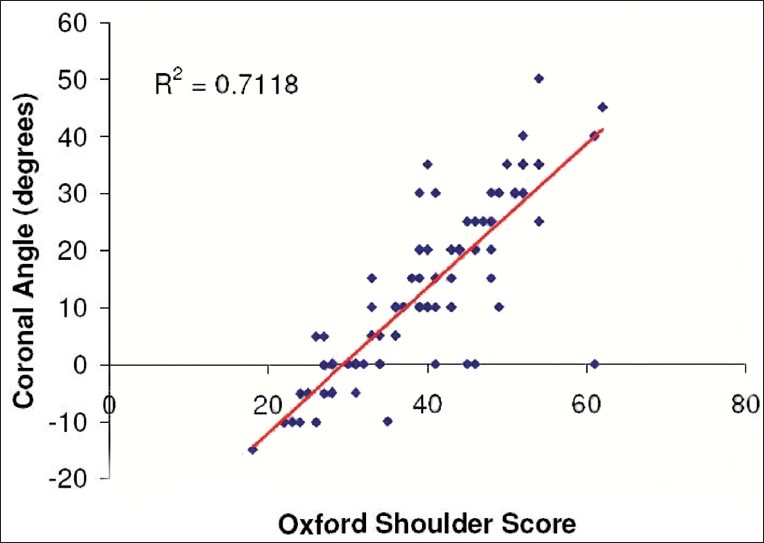
Correlation of the coronal plane angle with the Oxford shoulder score

Table 1 shows Pearson's correlation coefficients for components and totals for Constant-Murley and OSS with the coronal plane angle. Figures 3 and 4 illustrate the correlation between the total of the Constant and OSS, respectively, with the coronal plane angle. Table 2 illustrates the variation of the Coronal Plane angle, Constant-Murley score, and OSS according to the clinical diagnoses of shoulder pathology.

**Table 1:** Correlation of the coronal plane angle with the components of the Constant score and Oxford shoulder score

Score		Pearson coefficient
Constant	Total	0.90
	Subjective	0.84
	Pain	0.65
	Work	0.69
	Leisure	0.63
	Sleep	0.33
	Level of use	0.78
	Objective	0.76
	Flexion	0.60
	Abduction	0.64
	External rotation	0.63
	Internal rotation	0.57
OSS		0.84

**Table 2:** Patient case mix according to shoulder pathology and the associated mean scores for the coronal plane angle, Oxford shoulder score and Constant-Murley score

Pathology	Patient number	CPA	OSS	CS
Rotator cuff tear	54	+12.4°	39.9	35.5
Subacromial impingement	17	+9.8°	35.3	39.1
Osteoarthritis	7	+13.7°	41.1	32.7
Instabilty	4	Excluded due to apprehension		
Acromioclavicular dysfunction	8	+5.5°	33.2	41.2
Capsulitis	3	+21.1°	55.3	30.4
Other	7	+4.3°	28.5	46.7

CPA = coronal plane angle; OSS = Oxford shoulder score; CS = Constant-Murley score

## DISCUSSION

Codman introduced the concept of clinical outcomes to assess patient benefits after treatment.[4] It took many years before such assessment tools became widely used and standardized, thus allowing comparison of treatments across the literature. The Constant-Murley score has been widely accepted as the assessment tool for shoulder pathology and outcome across Europe.[2] However, despite low inter- and intraobserver error, it has been demonstrated to have wide, 95%, confidence limits of 16 to 20 points.[5] The Oxford Shoulder Questionnaire uses only subjective measures and correlates with the Constant Score,[3] which we have also demonstrated. Despite these multiple methods of assessment, they are time-consuming and rely on patient responses and multiple objective assessments.

The coronal plane angle is a simple and rapid assessment tool, using a single objective measurement of shoulder movement. It is applicable to most patients, with only nine exclusions in this cohort. The inability to get hand to head indicates poor function, as all five such patients scored poorly, with a Constant score of less than 10 and an OSS of greater than 55. The aforementioned scoring tools do not address the assessment of patients with instability, for which specific measures have been developed.[3,5] The coronal plane angle is also not applicable to patients with instability; as four were excluded from this study because of their apprehension, of which three had bilateral symptoms.

The coronal plane angle in the symptomatic shoulder was positive (anterior to the coronal plane = greater than zero) in the majority, with the mean being 11.3°. In contrast, the asymptomatic shoulder was negative (posterior to the coronal plane, less than zero) with a mean of −1.5°. This difference was statistically significant indicating that as a general marker of pathology it was a useful sign.

A significant correlation was observed between the Constant score total and the coronal plane angle (0.90). However, the correlation was not as significant for the components of the Constant score, which varied from 0.33 for sleep disturbance to 0.78 for the level of use. Surprisingly the external rotation component, which is similar to the coronal plane angle, did not have a high correlation (0.63). Overall the subjective components demonstrated a higher correlation than the objective components (0.84 and 0.76, respectively). It could be hypothesized that pain, as an isolated variable, would be a limiting factor in the assessment of the Coronal Plane angle, however, the correlation of pain and the angle was not as significant as some of the other variables [Table 1]. This would suggest that pain alone is not the solitary factor that results in a diminished coronal plane angle. These facts substantiate the use of the coronal plane angle in the assessment of shoulder pathology from both a subjective and objective point of view. Also, the OSS had a high correlation (0.84) with the coronal plane angle, which further substantiates the use of this tool in the assessment of shoulder pathology.

Measurement of the coronal plane angle does not differentiate between the causes of limited movement, whether due to pain, stiffness, weakness or any combination of these. We acknowledge that this study has not reviewed the intra- and interobserver variations in the assessment of the coronal plane angle. Nor does the angle take into account the disturbance of scapulothoracic rhythm, which would indirectly affect the Coronal Plane angle. This cohort of just over 100 shoulders is small, but representative of the average patient population typified by the high correlation between the Constant score and the OSS, which is consistent with the other published data.[2,3]

## CONCLUSION

The coronal plane angle is a single objective assessment and provides a simple alternative to shoulder assessment for a majority of patients. The correlation with both the Constant score and the OSS validates the use of this assessment tool in day-to-day practice, providing meaningful data while simplifying the collection of patient outcomes.
